# Diffusion tensor imaging along the perivascular space may reveal potential pathological mechanisms underlying disease progression in primary open-angle glaucoma patients

**DOI:** 10.3389/fneur.2025.1659200

**Published:** 2025-10-08

**Authors:** Yilei Chen, Shuyu Xiao, Lvyu Yan, Zhigang Gong, Yanwen Huang, Wenli Tan, Ying Yu

**Affiliations:** ^1^Department of Radiology, Shuguang Hospital Affiliated to Shanghai University of Traditional Chinese Medicine, Shanghai, China; ^2^Department of Ophthalmology, Shuguang Hospital Affiliated to Shanghai University of Traditional Chinese Medicine, Shanghai, China

**Keywords:** functional magnetic resonance imaging, primary open-angle glaucoma, glymphaticsystem, surface-based morphometry, diffusion tensor imaging along the perivascular space

## Abstract

**Purpose:**

This study investigates glymphatic system dysfunction in primary open-angle glaucoma (POAG) patients and explores its potential role in the progressive decline of visual function associated with the disease.

**Methods:**

This prospective study compared 47 primary open-angle glaucoma (POAG) patients and 50 healthy controls (HCs) using multimodal MRI, including DTI, T1/T2-weighted imaging, and resting-state fMRI. Group differences in brain morphometry, spontaneous activity, perivascular space (PVS) volume, and DTI-ALPS index were analyzed, with regression and mediation models exploring their relationships. Ocular parameters (intraocular pressure, RNFL thickness, cup-to-disc ratio, visual field) were correlated with fMRI findings, particularly PVS and ALPS metrics.

**Results:**

Compared to HCs, POAG patients exhibited significantly reduced cortical thickness, lower volume-wise Resting-state fMRI (Rs-fMRI) concordance (*p* < 0.001) and voxel-wise Rs-fMRI concordance (*p* < 0.05) in local intracranial regions, lower bilateral ALPS indices (*p* < 0.001), and higher volume fraction of the lateral ventricle body perivascular space (LVB-PVS) (*p* < 0.001). Linear regression models showed significant associations among left RNFL thickness, left ALPS index, LVB-PVS volume fraction, and cortical thickness of the left lingual gyrus (LING.L) (*p* < 0.05). Mediation analysis revealed that the left ALPS index partially mediated the associations between volume-wise Rs-fMRI concordance, cortical thickness of LING.L, and RNFL thickness. Furthermore, the ALPS index significantly mediated the relationship between LING.L cortical thickness and LVB-PVS volume fraction. However, no significant correlation was found between ALPS and the degree of visual field defect.

**Conclusion:**

The reduced ALPS index in POAG patients suggests impaired glymphatic clearance, which may impair metabolic clearance and contribute to RNFL damage, influencing disease progression.

## Introduction

Primary open-angle glaucoma (POAG), the second leading cause of blindness worldwide, currently affecting over 70 million individuals, with projections indicating that the number of cases will rise to 112 million by 2040, posing a significant global public health challenge (1–3).

Elevated intraocular pressure (IOP) is recognized as the most critical risk factor for POAG. However, factors such as complex vascular mechanisms, reduced cerebrospinal fluid pressure, the ocular-cranial pressure gradient, and immune factors are receiving increasing attention in the pathogenesis of glaucoma (4, 5). Prior work has shown progressive cortical thinning in POAG patients, starting in V5/MT + in mild cases and extending to V2 and V1 in more severe cases (6). There is accumulating evidence that glaucomatous neuropathy not only affects the retina, but also spreads to the central nervous system (CNS) (7). Therefore, POAG is recognized as a neurodegenerative condition similar to Alzheimer’s disease, affecting multiple brain regions, including the visual cortex, limbic system, and motor and sensory systems (8, 9). However, the underlying pathophysiology of this degeneration is yet to be determined.

Perivascular spaces (PVS) are fluid-filled spaces surrounding small perforating vessels in the brain. They play a key role in the brain’s fluid and glymphatic waste clearance systems (10, 11). The glymphatic system, a perivascular network responsible for removing metabolic wastes, plays a significant role in nervous system disorders, including Alzheimer’s disease. The glymphatic system has also been hypothesized to extend to the optic nerve and retina (12). The ocular glymphatic system having the similar clearance effect, may exist in the retina and optic nerve, which is supposed to be a part of the brain’s glymphatic system (13). Diffusion tensor imaging along the perivascular space (DTI-ALPS) can quantify differences in water molecule diffusion rates in various directions within the brain without requiring contrast agents. The ALPS index, derived using this methodology, offers a non-invasive way to evaluate glymphatic system function and serves as a promising neuroimaging biomarker for brain structural changes (10). It is still unclear whether glymphatic system contributes significantly to the development of glaucoma.

This study aims to evaluate the glymphatic system activity in patients with POAG using functional MRI and DTI-ALPS techniques. We explored the correlation between glymphatic activity, cortical structure, brain function, and Retinal Nerve Fiber Layer (RNFL) thickness. This study employs an interdisciplinary approach to scrutinize the alterations in the brain’s fMRI architecture and the fundus indicators of patients with primary POAG. This study investigates the correlation between impaired glymphatic clearance and POAG pathogenesis, focusing on its role in the progressive deterioration of visual function in glaucoma patients.

## Materials and methods

### Participants

This prospective observational study enrolled 47 POAG patients and 50 age−/sex-matched healthy controls (HCs) from Shuguang Hospital, Shanghai University of TCM (January 2023–June 2024). Three POAG participants were excluded (2 incomplete MRI scans; 1 structural brain abnormality). All participants were right-handed, free of neurological/psychiatric disorders, and provided written informed consent. The study protocol was reviewed and approved by the Ethics Committee of Shuguang Hospital, affiliated with Shanghai University of TCM (Ethics Review No. 2023–1,284-51-01), and the trial was registered on the Chinese Clinical Trial Registry, ChiCTR (registration number: ChiCTR2300070039).

#### POAG diagnostic criteria

POAG diagnosis adhered to the European Glaucoma Society criteria (2021), requiring open anterior chamber angles, glaucomatous optic neuropathy (enlargement of the cup-to-disc ratio, RNFL thinning on OCT), and reproducible visual field defects, with exclusion of secondary causes (14).

#### Key inclusion criteria

(1) Patients aged 35–70 years, regardless of sex; (2) Patients clinically diagnosed with POAG, currently receiving conventional intraocular pressure (IOP)-lowering treatment using eye drops; (3) Best-corrected visual acuity (BCVA) ≥ 0.1 in the study eyes; (4) Absence of other eye diseases that could cause visual field impairment or significantly affect visual field accuracy; (5) Willing to comply with the clinical research protocol.

#### Key exclusion criteria

(1) Patients with acute angle-closure glaucoma, congenital glaucoma, secondary glaucoma, or other types of glaucoma; (2) Patients with corneal diseases, severe cataracts (LOCS III ≥ 3), or vitreous opacities that obstruct fundus examination and data collection; (3) Other conditions affecting the RNFL, including a history of ophthalmic surgery, ocular or orbital trauma, optic atrophy, uveitis, diabetic retinopathy, any form of macular edema, or age-related macular degeneration; (4) Conditions significantly affecting MRI examination, including demyelinating diseases, autoimmune diseases, cardiovascular diseases (NYHA Class III/IV heart failure or Cr>2 mg/dL), and diabetes (fasting blood glucose concentration ≥7.1 mmol/L or using hypoglycemic drugs); (5) Patients receiving treatment with acetazolamide or other oral carbonic anhydrase inhibitors(CAIs); (6) Pregnant and lactating women; (7) Patients with severe mental illnesses such as depression, mania, schizophrenia, and other mental illnesses, Including people with neurodegenerative diseases such as Parkinson’s disease, Alzheimer’s disease, and multiple sclerosis; (8) Patients currently taking or requiring medications that could interfere with the evaluation of efficacy; (9) Individuals with contraindications to fMRI, such as claustrophobia or ferromagnetic implants, or those unable to cooperate with the examination.

All participants underwent standardized ophthalmic evaluation, slit lamp examination, computer optometry, IOP measurement (Non-contact intraocular pressure), perimetry and optic disc cube scans by two retinal specialists.

#### MRI acquisition

All imaging was conducted using a 3 T Siemens Skyra scanner equipped with a 32-channel head coil, adhering to the following protocols (The specific methods are detailed in the Supplementary materials):

#### Structural imaging

T1-MPRAGE: Repetition time (TR)/echo time (TE) = 2200/2.48 ms, resolution = 0.9 × 0.9 × 1.0 mm^3^. T2-TSE: TR/TE = 4000/103 ms, resolution = 0.6 × 0.6 × 6.0 mm^3^. Diffusion Tensor Imaging (DTI): 64 directions, b-value = 1,000 s/mm^2^, isotropic resolution = 2 mm. Resting-State fMRI: Echo-planar imaging (EPI) sequence, TR/TE = 2000/30 ms, slice thickness = 3.4 mm. Participants were instructed to remain awake with their eyes closed throughout the scan.

### Data processing

#### fMRI data preprocessing

Resting-state fMRI (Rs-fMRI) data were preprocessed using DPABI software^1^ within MATLAB 2013b.^2^ The first 10 time points were discarded, leaving 230 for analysis. Head motion correction was performed with slice timing and realignment, ensuring that motion in any direction remained below 2.0 mm or 2°. The functional images were co-registered and spatial normalized to Montreal Neurological Institute (MNI) space with 3 mm isotropic voxels. Nuisance covariate regression was performed to reduce low-frequency drifts, including white matter (WM), CSF signals, and 24 head motion parameters.

### Dynamic brain activity analysis

Dynamic brain activity was assessed using a sliding-window approach with a Hamming window. Five indices were calculated (Supplementary Figure S1): fALFF: Fractional amplitude of low-frequency fluctuations. ReHo: Regional homogeneity, reflecting local synchronization. VMHC: Voxel-mirrored homotopic connectivity, indicating inter-hemispheric connectivity. DC: Degree centrality, representing network centrality. GSC: Global signal correlation. Dynamic variability was quantified as the temporal standard deviation of each index. Rs-fMRI concordance across indices was evaluated using Kendall’s W at both voxel-wise and volume-wise levels.

### Structural analysis

This study employed voxel-based morphometry (VBM), deformation-based morphometry (DBM), and surface-based morphometry (SBM) analyses using MATLAB (r2013b) with the computational anatomy toolbox 12.8.1 (CAT12.8.1, http://www.neuro.uni-jena.de/cat/), an extension of SPM12.

T1-weighted images were processed using the CAT12 toolbox, employing the following methods: Voxel-Based Morphometry (VBM): Gray matter volume was estimated using DARTEL normalization and smoothed with an 8 mm Gaussian kernel. Surface-Based Morphometry (SBM): Cortical thickness and gyrification were computed, with smoothing kernels of 15–20 mm applied. Deformation-Based Morphometry (DBM): Jacobian determinant maps were generated and smoothed with an 8 mm kernel (The specific methods are detailed in the Supplementary materials-High-resolution T1 volumetric processing and imaging data analysis).

### Glymphatic system evaluation

#### DTI-ALPS index

**Figure 1:**
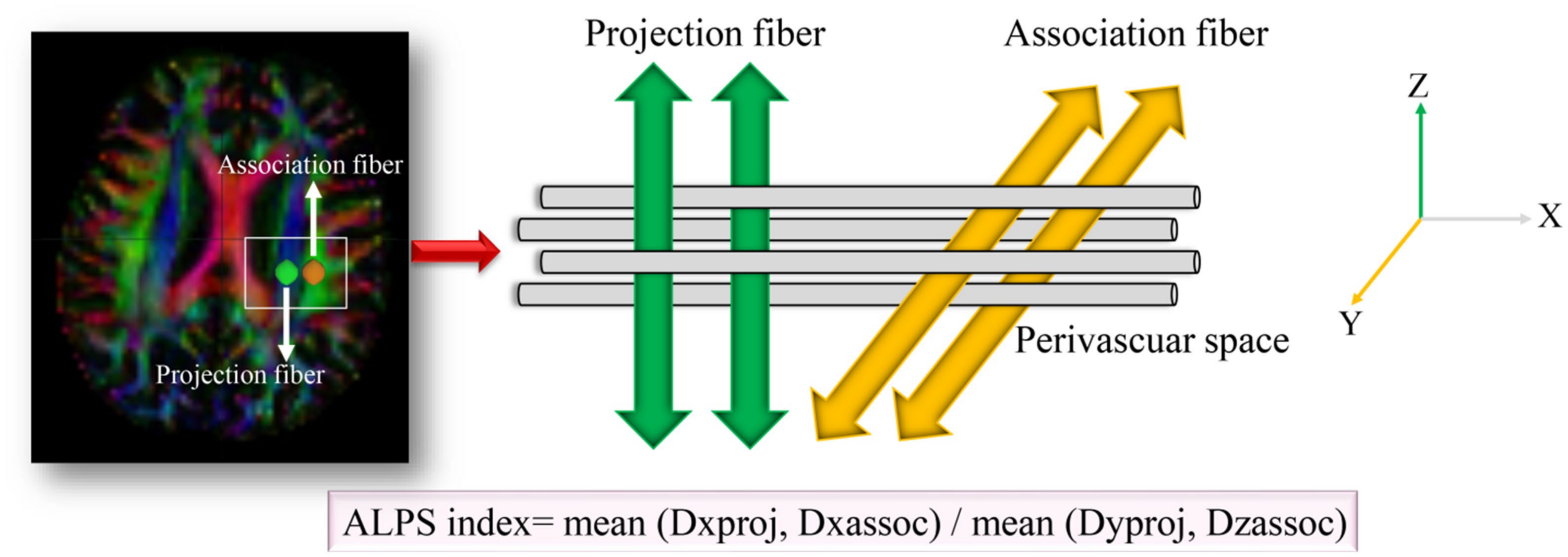
Method for acquiring the ALPS index analysis in this study. Dxxproj, diffusivity along the X-axis in the projection fiber; Dxxassoci, diffusivity along the X-axis in the association fiber; Dyyproj, diffusivity along the Y-axis in the projection fiber; Dzzassoci, the diffusivity along the Z-axis in the association fiber.

Diffusion-tensor images were preprocessed using DSI Studio graphic-user interface software version 10.15 (DSI Studio GUI; https://dsi-studio.labsolver.org/). Diffusion tensor imaging data were processed using DSI Studio, involving eddy current and motion correction, followed by fractional anisotropy (FA) map generation and alignment to a standard template. Regions of interest (ROIs) were placed at the level of the lateral ventricles, targeting projection and association fibers. The ALPS index was calculated as: ALPS-index = (Dxxproj + Dxxassoc)/(Dyyproj + Dzzassoc) (Figure 1). ROI analyses were independently conducted by two blinded neuroradiologists, achieving an intraclass correlation coefficient (ICC) > 0.85.

#### PVS volume quantification

PVS volumes were quantified from T2-weighted images using ITK-SNAP software (version 3.8; http://www.itksnap.org/). Segmentation targeted the basal ganglia, centrum semiovale, and lateral ventricle body. The volume fraction was calculated as: Volume fraction = PVS volume/(GM + WM volume). PVS volume Inter-rater reliability was confirmed, with an ICC > 0.90. The PVS volume fraction was defined as the ratio of the PVS volume to the total volume of GM and WM. GM and WM segmentation was performed using the CAT12.8.1.

#### Quantitative analysis of RNFL and ONH parameters

Optical coherence tomography (OCT) was performed using Zeiss Cirrus HD-OCT 5000 (Carl Zeiss Meditec, Inc., USA). Only high-quality images (signal strength ≥ 7) were included in the analysis. Each participant underwent a 200 × 200 optic disc cube scan to measure RNFL thickness (RNFLT). The scan protocol included a peripapillary circular scan with a diameter of 3.4 mm centered on the disc. The built-in software allowed for the mapping of the thickness data according to quadrant-by-quadrant and clock-hour analyses. Measure the RNFLT in the superior, inferior, nasal, and temporal quadrants of the optic disc and then take the average.

### Statistical analysis

Continuous variables were presented as mean ± standard deviation (SD). Normality was assessed using the Shapiro–Wilk test and visual inspection of histograms. Clinical data between groups were compared using a two-sample t-test. Statistical analyses were performed using SPSS Statistics software (version 25), with statistical significance defined as *p* < 0.05. A two-sample t-test, adjusted for age, sex, and years of education as covariates, was used to evaluate differences in global structural morphometry between patients with POAG and HCs, with statistical significance set at *p* < 0.05. Differences in cortical thickness, sulcal depth, cortical complexity, gyrification index, and GM volume (assessed by DBM and VBM) between groups were assessed using two-sample t-tests, adjusting for age, sex, and education years. Additionally, TIV was included as a covariate in VBM models. The false discovery rate (FDR) was employed for multiple comparison correction, applying a voxel-level threshold of *p* < 0.001 and a cluster-level threshold of *p* < 0.05.

Volume-wise Rs-fMRI concordance differences between patients with POAG and HCs were compared using voxel-based two-sample t-tests, with sex, age, and education years as nuisance covariates. Between-group differences in voxel-wise Rs-fMRI concordance were tested similarly. Multiple comparison correction was performed using Gaussian random field theory (GRF), with voxel-level *p* < 0.001 and cluster-level *p* < 0.05 (two-tailed). Baseline statistics were derived from eye examination data, analyzed using an independent-sample t-test.

The ALPS-index and PVS volume were compared using a two-sample t-test or Mann–Whitney U test, with age, sex, and education years as covariates and statistical significance set at *p* < 0.05. Inter-observer agreement for ALPS-index and PVS volume was evaluated using intraclass correlation coefficients (ICCs) across all participants (15) (range and correlation: 0.00–0.20, poor; 0.21–0.40, fair; 0.41–0.60, moderate; 0.61–0.80, good; and 0.81–1.00, excellent).

Linear regression analyses were conducted to examine the associations between clinical indicators and PVS volumes, cortical morphological alterations, Rs-fMRI concordance indices, and DTI-ALPS, with adjustments for age, sex, and years of education. To investigate sequential changes in PVS volumes, cortical morphological alterations, Rs-fMRI concordance indices, and DTI-ALPS, all data were normalized to a 0–1 scale. The logistic curves were used to model the probability of a certain outcome as a function of continuous predictors. Non-linear curve fitting models were applied to explore the relationships between these variables and RNFLT using OriginPro 2022 (OriginLab, Northampton, MA, USA). On the other hand, the mediation analysis examines whether the ALPS-index acts as a mediator between two other variables. Mediation analysis examined relationships among PVS volumes, cortical morphology, Rs-fMRI concordance indices, DTI-ALPS, and RNFLT. In this analysis, volume-wise Rs-fMRI concordance and cortical thickness of the left lingual gyrus (LING.L) were independent variables, RNFLT and LVB-PVS volume fraction were dependent variables, and the ALPS index served as the mediator, with age, sex, and years of education as covariates. The mediation analysis measures the indirect effect of the independent variable (e.g., cortical thickness) on the dependent variable (e.g., RNFLT) via the mediator (ALPS-index). Following established research methodologies (16), the mediation model was computed using PROCESS v3.4 in SPSS 25, which involved the application of model 4 (simple mediation model) from the macro created by Andrew Hayes.^3^ The mediation effect was considered significant if the 95% confidence interval (CI) for the indirect effect did not include zero.

## Results

### Demographic and clinical characteristics

Table 1 presents the demographic and clinical characteristics of the study participants, including 47 patients with POAG and 50 HCs. Briefly, the two groups showed no significant differences in age (t = −1.703, *p* = 0.092), sex (χ^2^ = 0.457, *p* = 0.499), education years (t = −1.39, *p* = 0.441), or equivalent spherical lens (R: t = −0.30, *p* = 0.922; L: t = 0.71, *p* = 0.872). In contrast, IOP (*p* = 0.001), RNFL (*p* = 0.019), cup-to-disc ratio (CDR) horizontal (*p* = 0.018), CDR vertical (*p* = 0.008), R/D min (*p* = 0.003), and R/D angle (*p* = 0.048) were significantly different between the two groups. No significant difference was found in the optic disc area (*p* = 0.383) between the two groups. However, the optic cup area was significantly different (*p* = 0.001) between the two groups (Table 2). There was no statistically significant difference between the left and right eyes in POAG (Table 3).

**Table 1:** Demographics of participants.

Characteristics	POAG (*n* = 47)	HCs (*n* = 50)	*p* value
Age (years)	52.90 ± 10.10	49.50 ± 9.09	0.092
Sex (female/male)	25/22	30/20	0.499
Education (years)	16.95 ± 1.88	17.55 ± 1.80	0.441
Disease duration (years)	6.04 ± 4.50	-	-

POAG, primary open-angle glaucoma; HCs, healthy controls.

**Table 2:** Differences between patients with POAG and HCs in IOP, RNFL, C/D, R/D, optic disc area, and optic cup area.

Characteristics (Mean ± SD)	POAG (*n* = 47)	HCs (*n* = 50)	*p* value
RNFL	67.115 ± 16.019	104.787 ± 12.543	0.019
IOP (mmHg)	15.390 ± 3.684	15.583 ± 1.603	0.001
CDR horizontal	0.642 ± 0.111	0.557 ± 0.145	0.018
CDR vertical	0.664 ± 0.111	0.519 ± 0.171	0.008
R/D min	0.076 ± 0.055	0.143 ± 0.071	0.003
R/D angle	124.618 ± 92.455	165.890 ± 115.470	0.048
Optic disc area	2.317 ± 0.905	2.319 ± 0.555	0.383
Optic cup area	1.009 ± 0.561	0.698 ± 0.479	0.001

These data were shown as the mean value of right and left eyes in each patient or HC. POAG, primary open-angle glaucoma; HCs, healthy controls; RNFL, retinal nerve fiber layer; CDR, cup-to-disc ratio; R/D, rim-to-disc ratio; SD, standard deviation.

**Table 3:** Differences between left and right eyes in IOP, RNFL, C/D, R/D, optic disc area, and optic cup area in POAG.

Characteristics (Mean ± SD)	Right (*n* = 47)	Left (*n* = 47)	*p* value
IOP (mmHg)	15.619 ± 4.176	16.257 ± 3.877	0.467
RNFL	66.778 ± 16.752	70.930 ± 19.438	0.285
CDR horizontal	0.640 ± 0.113	0.644 ± 0.109	0.971
CDR vertical	0.660 ± 0.113	0.668 ± 0.109	0.757
R/D min	0.078 ± 0.063	0.075 ± 0.041	0.810
R/D angle	122.070 ± 64.689	129.079 ± 120.242	0.750
Optic disc area	2.384 ± 1.089	2.318 ± 0.712	0.750
Optic cup area	1.051 ± 0.651	1.001 ± 0.455	0.690

RNFL, retinal nerve fiber layer; CDR, cup-to-disc ratio; R/D, rim-to-disc ratio; SD, standard deviation.

### Global structural morphometry and cortical morphology alterations in patients with POAG

**Figure 2:**
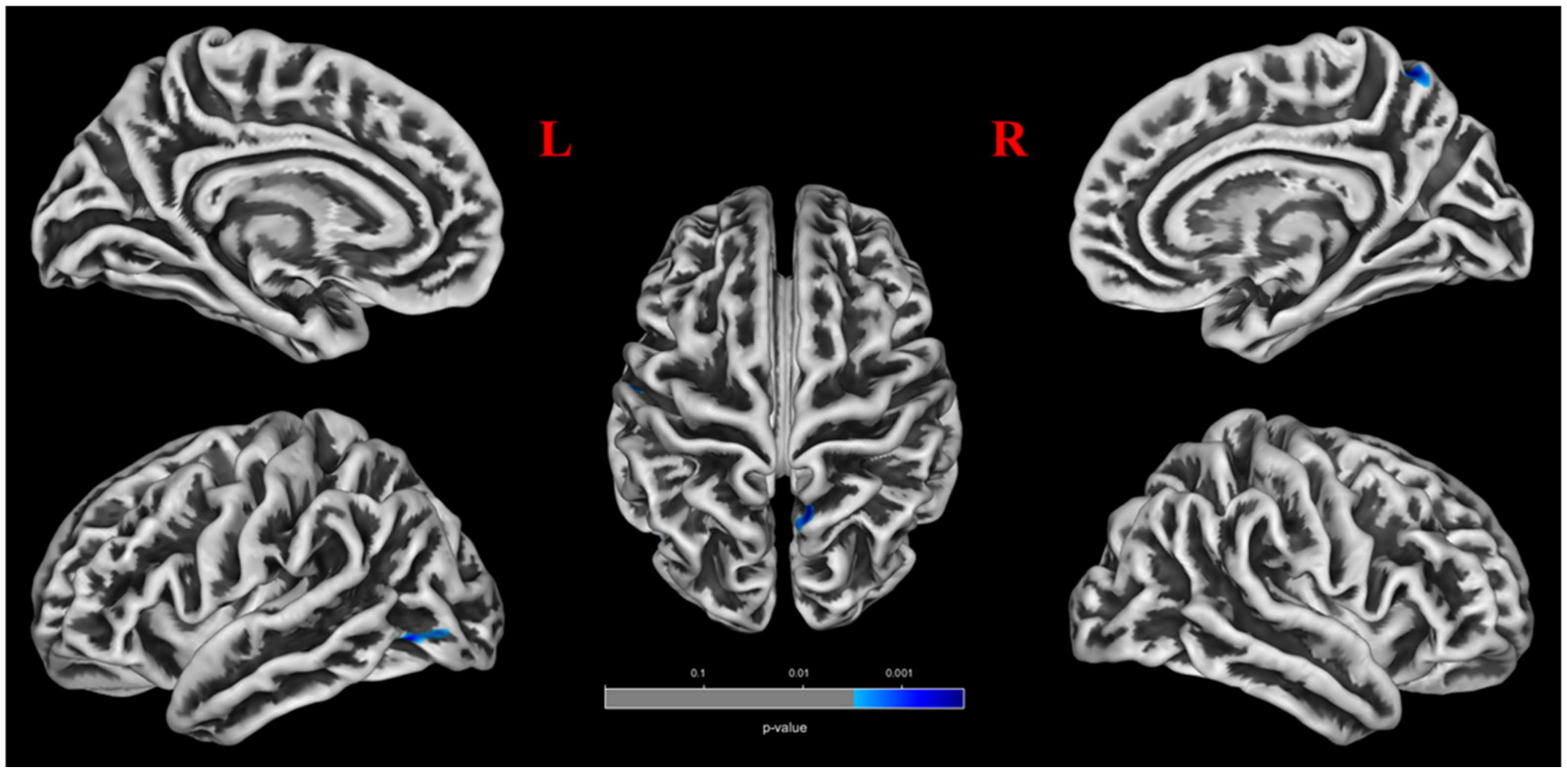
Comparison of the cortical thickness of patients with POAG and HCs.

No significant differences were found between patients with POAG and HCs in global GM volume, WM volume, and CSF volume after adjusting for TIV, age, sex, and education years (Figure 2). However, patients with POAG exhibited reduced cortical thickness in three clusters compared to HCs, including the left middle occipital gyrus (MOG.L), left postcentral gyrus (PoCG.L), and right precuneus (PCUN.R) (*p* < 0.05, FDR corrected) (Figure 2, Table 4). No clusters of sulcal depth and increased cortical thickness were observed in patients with POAG compared to HCs. Additionally, no significant differences were found in the whole-brain VBM and DBM analyses between patients with POAG and HCs (*p* > 0.05, FDR corrected).

**Table 4:** Clusters showing significantly changed cortical morphology in patients with POAG compared to HCs.

Measurement	Brain region	Hemisphere	Peak MNI coordinates			Cluster size (voxels)	Peak intensity
			X	Y	Z		
SBM							
Cortical thickness	middle occipital gyrus	L	−35	−85	14	329	4.234
	postcentral gyrus	L	−45	−25	49	153	3.456
	precuneus	R	12	−58	45	175	4.023

SBM, surface-based morphometry; POAG, Primary open-angle glaucoma; HCs, healthy controls. The cool color regions representing significantly thinner cortical regions in patients with POAG (FDR, voxels *p* < 0.001, clusters *p* < 0.05). The color bar indicates the voxel-wise *p*-value. POAG, Primary open angle Glaucoma.

### Volume-wise and voxel-wise Rs-fMRI concordance alterations in patients with POAG

**Figure 3:**
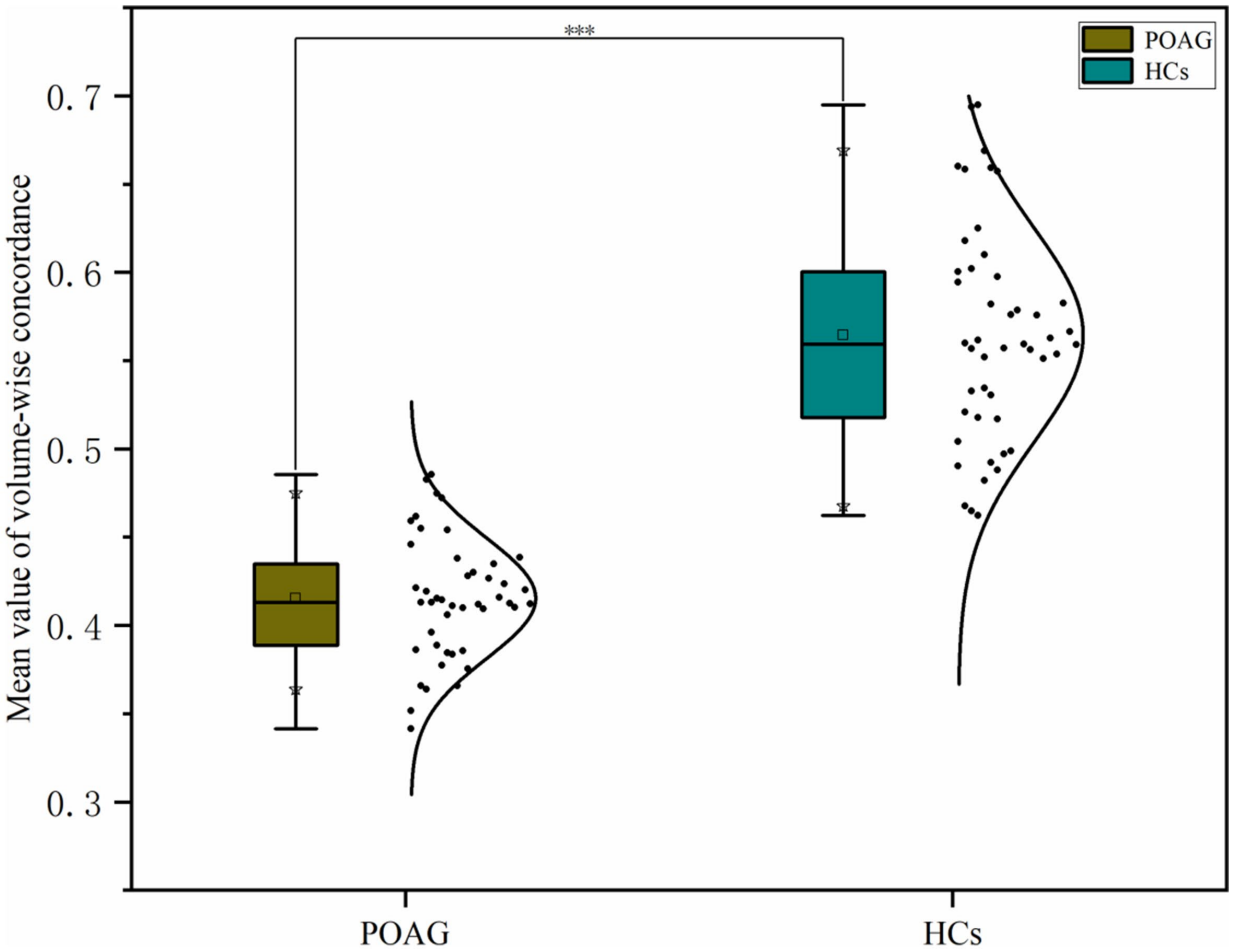
Comparison of mean volume-wise Rs-fMRI concordance indices between patients with POAG and HCs. “***” Indicates *p* < 0.001. POAG, Primary open angle Glaucoma; HCs, healthy controls.

**Figure 4:**
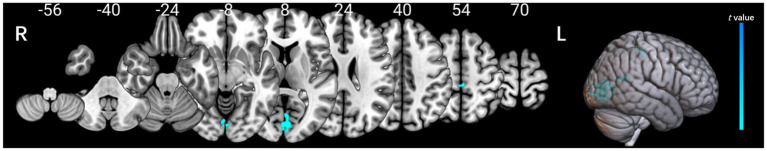
Comparison of the voxel-wise Rs-fMRI concordance between patients with POAG and HCs.

Patients with POAG exhibited a significantly decreased mean value of volume-wise Rs-fMRI concordance compared to HCs (*p* < 0.001) (Figure 3). Significant differences in voxel-wise Rs-fMRI concordance between patients with POAG and HCs are shown in Figure 4. We found decreased voxel-wise Rs-fMRI concordance in multiple regions, including bilateral lingual gyrus (LING.L/R), calcarine fissure and surrounding cortex, and left posterior cingulate gyrus (*p* < 0.001).

The cool color regions representing significantly reduced Rs-fMRI concordance in patients with POAG (GRF, voxels *p* < 0.001, clusters *p* < 0.05). POAG, Primary open angle Glaucoma; HCs, healthy controls.

### Comparison of PVS volume fraction between patients with POAG and HCs

**Figure 5:**
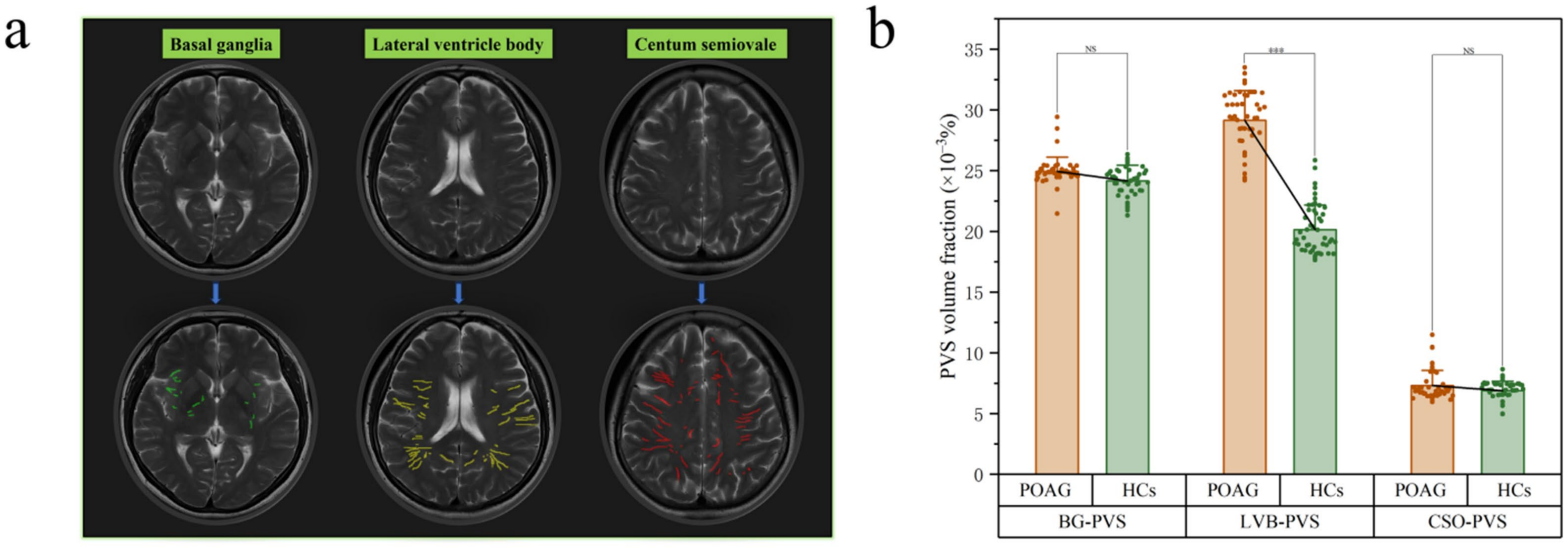
Example of PVS segmentation results in a patient with POAG. This picture illustrates T2 weighted images and PVS mask in basal ganglia (green), level of lateral ventricle body (yellow), and the centum semiovale (red). **(a)** Differences in PVS volume fraction among patients with POAG and HCs. **(b)** A statistically significant difference was observed between the POAG and healthy control (HC) groups in the LVB-PVS comparison (*p* < 0.001). “***” indicates *p* < 0.001. “NS” denotes no statistically significant difference. POAG, Primary open angle Glaucoma; HCs, healthy controls; PVS, perivascular space; BG, basal ganglia; LVB, Lateral ventricle body; CSO, centum semiovale. The upper column is the original T2WI transverse image **(a)**. The lower column is the PVS volume in three levels based on the ITK-SNAP software package **(a)**.

In patients with POAG, the ICCs for BG-PVS, CSO-PVS, and LVB-PVS were 89.1% (95% CI: 0.86–0.93, *p* < 0.001), 92.5% (95% CI: 0.89–0.94, *p* < 0.001), and 94.1% (95% CI: 0.90–0.96, *p* < 0.001), respectively, compared to 95.5% (95% CI: 0.91–0.97, *p* < 0.001), 91.2% (95% CI: 0.87–0.94, *p* < 0.001), and 93.1% (95% CI: 0.89–0.95, *p* < 0.001) in HCs (Supplementary Figures S2–S7). Figure 5a illustrates an example of a PVS segmentation result image from a single patient with POAG. Patients with POAG had significantly higher LVB-PVS volume fraction than HCs (*p* < 0.001), while no significant differences were found in CSO-PVS or BG-PVS volume fraction between the two groups (Figure 5b). No significant difference was found between the POAG and healthy control (HC) groups for BG-PVS and CSO-PVS.

### Differences in ALPS-index between patients with POAG and HCs and visual field deficit severity subgroup analysis

**Figure 6:**
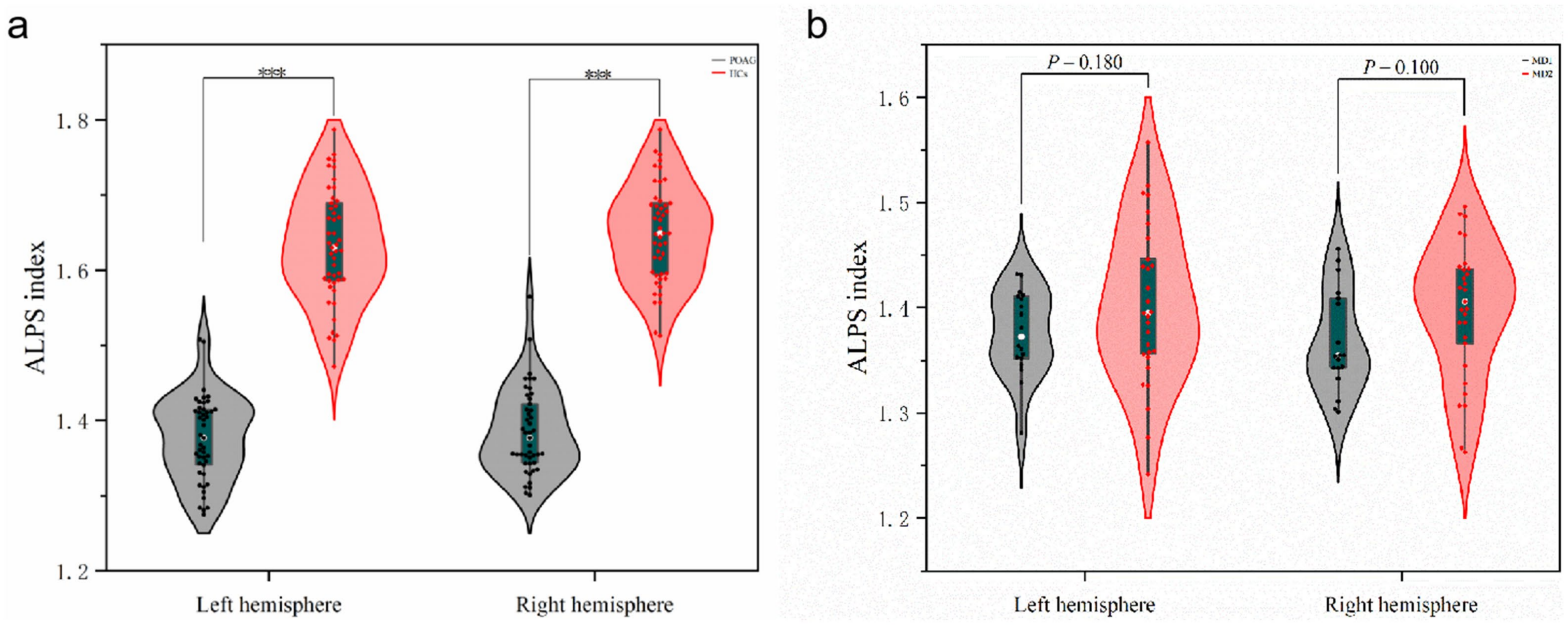
Differences in ALPS-index between patients with POAG and HCs and visual field deficit severity subgroup analysis. **(a)** The differences of left ALPS-index and right ALPS-index between patients with POAG and HCs. “***” Indicates *p* < 0.001. POAG, Primary open angle Glaucoma; HCs, healthy controls. **(b)** The differences of left ALPS-index and right ALPS-index between two subgroups. MD1, mild and moderate visual field defect group; MD2, severe visual field defect group.

For the left ALPS-index (ICC = 0.876, 95% CI: 0.85–0.93, *p* < 0.001) and the right ALPS-index (ICC = 0.865, 95% CI: 0.84–0.92, *p* < 0.001) (Supplementary Figures S8–S11), the inter-observer reliability was excellent in patients with POAG. Similarly, for HCs, the inter-observer reliability was very good, with ICCs of 86.2% (95% CI: 0.83–0.91, *p* < 0.001) and 87.1% (95% CI: 0.84–0.93, *p* < 0.001) for the left and right ALPS-indices, respectively. The bilateral ALPS-index was significantly lower in patients with POAG compared to HCs (*p* < 0.001) (Figure 6a). Subgroup analysis based on visual field deficit severity (mild: MD > −6 dB, moderate: MD between −6 and −12 dB, severe: MD < −12 dB) were also conducted. We reclassified the cases in our dataset, grouping mild and moderate visual field deficits together(MD1 group:18 cases) and designating severe deficits as a separate group (MD2 group:29 cases) to ensure adequate sample sizes for comparison. However, no significant differences of ALPS-index were found in the two subgroups (Figure 6b).

### Relation between PVS volumes, cortical morphology alterations, Rs-fMRI concordance indices, ALPS-index, and clinical indicators

**Figure 7:**
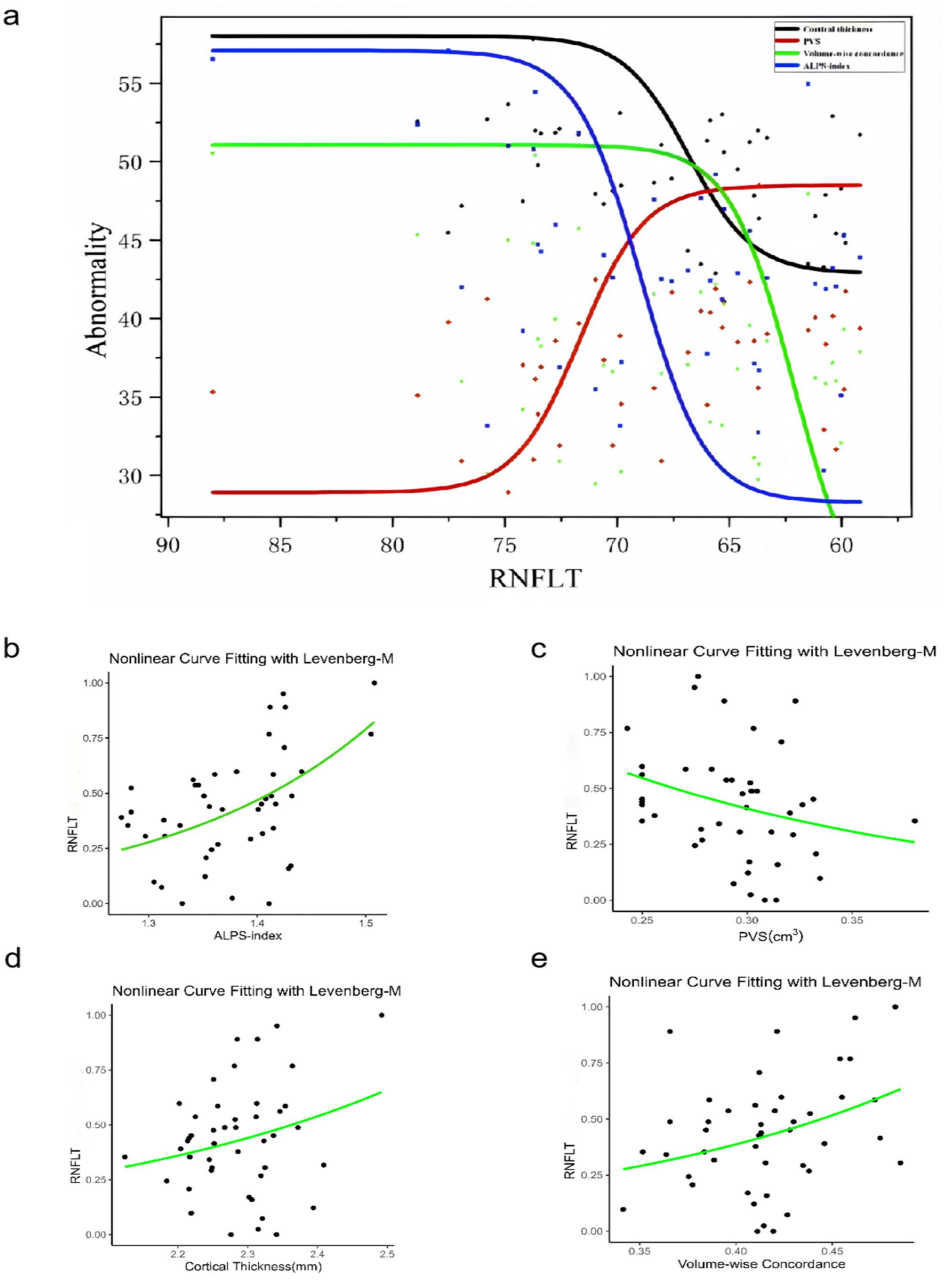
The relationship among the PVS volume fraction, cortical morphology alterations, Rs-fMRI concordance indices, DTI-ALPS, and RNFLT in patients with POAG. The results of the logistic curve fitting model for the ALPS-index **(b)** (*p* =0.000212***), PVS volume fraction **(c)** (*p* = 0.0562), cortical thickness **(d)** (*p* =0.0904), volume-wise Rs-fMRI concordance **(e)** (*p* = 0.0194*), and RNFLT in patients with POAG. Combined fitting curves from the ALPS-index, PVS volume fraction, cortical thickness, volume-wise Rs-fMRI concordance **(a)**. “Abnormality” indicates the dependent variable is standardized by the fitted model. ALPS, analysis along the perivascular space; RNFLT, left retinal nerve fiber layer thickness; PVS, perivascular space; POAG, primary open-angle glaucoma.

Using linear regression models, the left RNFLT exhibited significant associations with the left ALPS-index (β = 0.234, *p* = 0.014), the LVB-PVS volume fraction (β = −0.215, *p* = 0.013), cortical thickness of the left lingual gyrus (LING.L, β = 0.204, *p* = 0.020), and volume-wise Rs-fMRI concordance (β = 0.284, *p* = 0.005). No analogous associations were observed in the right eye (Table 5). In contrast, the left cup/disc ratio (C/D) was solely correlated with the LVB-PVS volume fraction (β = 0.305, *p* = 0.030). No significant relationships were identified between other clinical parameters and PVS volumes, cortical morphological changes, Rs-fMRI concordance indices, or ALPS-index. A logistic curve-fitting analysis revealed distinct trajectories for RNFLT in relation to various parameters (Figure 7a). Specifically, RNFLT increased with rising ALPS-index (Figure 7b) and volume-wise Rs-fMRI concordance (Figure 7e) (*p* < 0.001 and *p* < 0.05, respectively). Similarly, RNFLT fitting curves for cortical thickness (Figure 7d) of the LING.L showed a gradual increase followed by a plateau. Conversely, the fitted curve for RNFLT declined as LVB-PVS volume fraction increased (Figure 7c). The trajectory of ALPS indices closely mirrored that of cortical thickness but demonstrated a delayed onset. The curve-fitting model suggested that turning points for changes in ALPS indices occurred between those for volume-wise Rs-fMRI concordance, cortical thickness and alterations in PVS volume fractions. This temporal sequence underscores the potential mediatory role of the ALPS-index, as further supported by statistical mediation analysis.

**Table 5:** Regression analysis among PVS volumes, cortical morphology alterations, Rs-fMRI concordance indices, ALPS-index, and clinical indicators.

Parameter	RNFLT(L)		RNFLT(R)		C/D(L)		C/D(R)	
	Coefficient	*p* value	Coefficient	*p* value	Coefficient	*p* value	Coefficient	*p* value
left ALPS-index	0.234	0.014*	0.236	0.109	0.305	0.030*	0.297	0.101
LVB-PVS volume fraction	−0.215	0.013*	−0.164	0.065	−0.187	0.181	−0.121	0.512
cortical thickness in MOG.L	0.204	0.020*	0.105	0.099	0.113	0.284	0.118	0.471
cortical thickness in PoCG.L	−0.116	0.219	−0.250	0.186	−0.076	0.300	−0.142	0.160
cortical thickness in PCUN.R	0.090	0.303	0.032	0.834	0.058	0.513	0.194	0.236
volume-wise Rs-fMRI concordance	0.284	0.005*	0.178	0.054	0.326	0.056	0.171	0.323

### Mediation analysis based on PVS volumes, cortical morphology alterations, Rs-fMRI concordance indices, ALPS-index, and RNFLT

**Figure 8:**
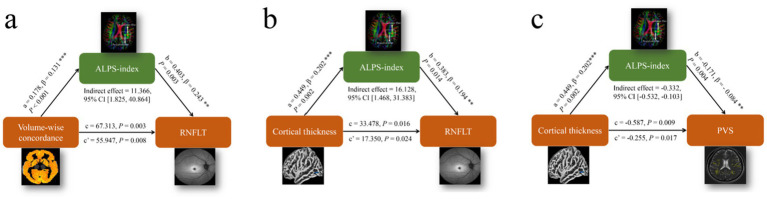
Results of the mediation analysis on PVS volumes, cortical morphology alterations, Rs-fMRI concordance indices, ALPS-index, and RNFLT. **(a)** Mediation analysis between volume-wise Rs-fMRI concordance (X) and RNFLT (Y), with ALPS-index as mediator (M). **(b)** Mediation analysis between cortical thickness of LING. L (X) and RNFLT (Y), with ALPS-index as mediator (M). **(c)** Mediation analysis between cortical thickness of LING. L (X) and LVB-PVS volume fraction (Y), with ALPS-index as mediator (M). ALPS, analysis along the perivascular space; LING. L, left lingual gyrus; RNFLT, left retinal nerve fiber layer thickness; PVS, perivascular space.

Mediation analysis was performed in patients with POAG to investigate the relationships among PVS volumes, cortical morphology alterations, Rs-fMRI concordance indices, DTI-ALPS, and RNFLT. Volume-wise Rs-fMRI concordance and cortical thickness of LING.L were designated as the independent variables (X), RNFLT and LVB-PVS volume fraction as the dependent variables (Y), and the left ALPS-index as the mediating variable (M). The mediation analysis revealed that the left ALPS-index partially mediated the relationship between volume-wise Rs-fMRI concordance and RNFLT (indirect effect = 11.366, 95% CI: 1.825–40.864), based on 5,000 bootstrap samples and adjusted for age, sex, and education years (Figure 8a). The ALPS-index also exhibited significant mediation effects between the cortical thickness of LING.L and RNFLT (indirect effect = 16.128, 95% CI: 1.468–31.383) (Figure 8b), contributing to 16.89 and 48.17% of the total effects, respectively. Additionally, the ALPS-index significantly mediated the relationship between the cortical thickness of LING.L and LVB-PVS volume fraction (indirect effect = −0.332; 95% CI: −0.532–-0.103) (Figure 8c), accounting for 56.56% of the total effects in this relationship.

## Discussion

This study provides novel evidence linking primary POAG to both central neurodegeneration and impaired glymphatic clearance. High-resolution 3D T1-weighted imaging revealed significant abnormalities in brain structure volume and thickness in patients with POAG. The specific regional cortical thinning was evident in POAG, particularly in the MOG.L, PoCG.L, and PCU.R (*p* < 0.05, FWE corrected). The MOG.L is crucial for visual information processing, while the precuneus, located medially in the parietal lobe, is involved in visuospatial processing (17). Furthermore, the precuneus serves as a pivotal hub within the default mode network, playing a crucial role in cognitive processes including episodic memory and self-referential processing. In Alzheimer’s disease, the precuneus is considered a central site for tau pathology deposition and neuroinflammation (18). Our study also found a significant correlation between left eye RNFL thickness and MOG.L cortical thickness (*p* < 0.05), suggesting a link between optic nerve and visual cortical changes in POAG patients, consistent with previous research (19).

Previous studies have demonstrated morphological, functional, and metabolic damage to nervous system sites in patients with POAG (20). Haykal were the first to apply NODDI to study white matter degeneration in the visual pathway in POAG (21), highlighting the importance of neuroimaging biomarkers in early detection, which aligns with the purpose of using the ALPS index in our study. Both aim to provide information about the microstructure of white matter tracts to help uncover structural changes in neurodegenerative diseases. However, unlike their study, which primarily focused on white matter integrity, we expand on these findings by exploring the relationship between ALPS and ocular structural changes, offering a more comprehensive view of the disease’s pathophysiology. In this study, a sliding window approach was employed to systematically compute five resting-state functional MRI (Rs-fMRI) metrics, including fALFF, ReHo, VMHC, DC, and GSCorr. To capture dynamic features, a Hamming window was used to segment the whole-brain BOLD signal, and the aforementioned metrics were calculated within each time window. This method aimed to comprehensively characterize the dynamic changes in local brain activity features and inter-metric consistency, instead of individual index and group analysis. Rs-fMRI concordance does not reflect heterogeneity within the glaucoma diagnosis per se, but rather the degree of functional coordination across dynamic brain activity indices. Reduced +Rs-fMRI concordance thus suggests a disruption of large-scale functional integration, complementing the univariate findings and providing a multidimensional view of POAG-related brain dysfunction. Our findings showed a significantly lower mean volume Rs-fMRI concordance in patients with POAG compared to HCs, indicating disrupted synchronization of brain networks in these patients.

The primary anatomical component of the glymphatic system is the Virchow-Robin space, or perivascular space, responsible for clearing brain metabolites. Recent studies on glaucoma have increasingly highlighted glymphatic function, particularly focused on the obstruction of glymphatic drainage at the cribriform plate. The imbalance between cerebrospinal fluid and intraocular pressure deforms the sieve plate, constricting blood vessels and nerve spaces, which may also impair glymphatic function. Experimental studies suggest that deformities in the cribriform plate impair the glial-lymphatic system’s clearance, reducing the removal of intraocular metabolites such as β-amyloid and other neurotoxins, which contribute to glaucoma progression (13, 22–24). Furthermore, a recent research discovered Aβfrom the brain can flow into the eyes along the optic nerve through CSF, causing retinal degeneration (25). This phenomenon may explain why some patients continue to experience visual field progression despite adequate intraocular pressure control. Further investigation using functional MRI techniques may provide insights into the intracranial mechanisms underlying this observation.

The PVS plays a crucial role in intracerebral glymphatic circulation, and its enlargement may indicate fluid flow interruption and stagnation. Our study revealed that the increased PVS volume in POAG patients (*p* < 0.001) was negatively correlated with the average thickness of the nerve fiber layer in the left eye and positively correlated with the left cup/disk ratio (Table 5). This suggests that damage to the optic nerve fiber layer is associated with the expansion of the cerebral glymphatic return space in POAG patients.

In the DTI-ALPS inspection to assess the glymphatic system. The ALPS-index has emerged as a novel biomarker for assessing conditions associated with the glymphatic system (26). In our study, we observed that the ALPS-index was significantly lower in patients with POAG compared to HCs (Figure 6a), and it correlated positively with the left eye RNFLT (*p* < 0.05, Table 5). We also employed an intermediary analytical approach that mediates the relationship between cortical thinning, PVS alterations, and RNFLT, underscoring its potential to link cortical and glymphatic changes with clinical indicators of glaucoma severity (Figure 8). Carvalho highlighted neuroplasticity in the visual cortex of glaucoma patients (27). Our results provide a structural basis for this by identifying specific white matter changes linked to visual function. This aligns with their findings and offers a clearer view of the underlying structural damage. The mediation analysis further revealed that ALPS-index may significantly links cortical atrophy to RNFL thinning, suggesting a “retina-brain-glymphatic” axis in POAG pathogenesis.

In our study, there was no difference between subgroups with varying degrees of visual field injury, suggesting that ALPS may better reflect structural changes than visual function impairment (Figure 6b). But in another recent study, researchers found in NTG (normal tension glaucoma) patients, the right ALPS indexes of NTG patients were positively correlated with the MD values of the left eyes (28). The ALPS index may serve as a promising biomarker linking ocular and central nervous system pathology in primary POAG, potentially enhancing our understanding of the disease’s underlying pathophysiological mechanisms. Demaria found significant functional changes in local brain networks, especially in visual-related regions and the prefrontal cortex, in POAG patients with binocular visual field defects (29). While their study focuses on functional indicators, ours targets structural changes, offering a new perspective for future research.

Despite the prospective design of this study, it has some limitations. Firstly, current measures primarily reflect periventricular glymphatic activity; whole-brain assessments require advanced protocols. And the role of local glymphatic dysfumction of the eye remains to be futher studied. Other methods, such as gBOLD-CSF coupling and free water imaging (15, 30), may also assess glymphatic activity. Integrating these methods could offer a more complete evaluation of brain lymphatic function—a key objective for future studies. Secondly, our study employed a cross-sectional design with a small sample size. Longitudinal studies are needed to establish temporal relationships between impaired glymphatic clearance and disease progression. Thirdly, the relationship between changes in glymphatic function and ocular indicators revealed in our study was mostly concentrated on the left side, and the specific reason is unknown. Some scholars pointed out that brain function is lateralized, and many basic cognitive functions, neurophysiological functions, and information exchange between the left and right hemispheres of the human brain are asymmetrical (31). Coincidentally, a recent study also highlighted ALPS’s role in glymphatic function in NTG patients, measuring only the correlation between changes in the right ALPS and the left eye visual field. The inconsistency of functional structure between the two sides is indicated (28). Therefore, we speculate whether the difference in the exploration of the correlation between the left and right brains and visual function is related to the lateralized brain functions. The patients we included this time are all right-handed, and the left brain is the dominant brain, which may also have an impact on the result. Further research and large samples are needed in the future.

Moreover, the DTI-ALPS index was calculated using a voxel size of 2 × 2 × 3.4 mm, which is consistent with standard clinical DTI protocols. While higher resolutions (e.g., 1 mm isotropic) could reduce partial volume effects, our resolution balances spatial detail, SNR, and scan time. Future studies may explore advanced diffusion techniques to further refine PVS characterization.

We tested only the direct and mediation models involving the ALPS-index, given the study scope and data limitations. Other models (e.g., moderated or multilevel mediation) were not examined. For this limitation, future studies should investigate alternative mediation models to provide a more comprehensive understanding of the complex relationships among these variables. The biological interpretation of the DTI-ALPS index remains debated. Although often considered an indirect marker of glymphatic function, it may also reflect broader white matter microstructural properties and is sensitive to methodological factors. Thus, its interpretation should be made with caution, and further studies are needed to delineate the underlying neurobiological processes.

We recognize the importance of open science and reproducibility. Raw data and analysis pipelines will be available on GitHub^4^ upon publication. This includes preprocessing pipelines, statistical scripts, and visualization codes. Data sharing will adhere to institutional and ethical guidelines, with de-identified data available upon request. Future work should validate our framework in independent, international cohorts to enhance generalizability and enable cross-population comparisons. Open data availability makes our methods accessible to researchers worldwide, enhancing the robustness and generalizability of the findings.

In summary, our study examined changes in brain structure, function, and glymphatic function in patients with POAG. It is apparent that POAG is associated with structural and functional changes in specific intracranial regions. There may be some controversy as to whether the DTI-Alps technique directly represents the colloidal lymphatic system. While our current findings are not exclusively localized to visual processing regions, these preliminary observations still provide valuable insights and important directions for future investigation. Professor Iliff mentioned that “Changes in the measure are seen under conditions where glymphatic function are impaired—so while we do not have a strong handle on the exact nature of the signal it seems to work at some level (26). The non-invasive ALPS-index may serve as a sensitive neuroimaging biomarker in POAG patients, advancing early screening, precise diagnostics, and timely therapeutic interventions. Future research should focus on large-sample, multi-center prospective cohort studies using machine learning and deep learning methods to further explore and analyze MRI data and ocular manifestations.

## Conclusion

Our multimodal MRI approach reveals that POAG involves both impaired glymphatic clearance and cortico-visual network degeneration. The DTI-ALPS index, despite methodological limitations, emerges as a promising biomarker for bridging ocular and central nervous system pathology. Notably, the ALPS-index is pivotal in connecting cortical atrophy, reduced Rs-fMRI concordance, and PVS expansion with the loss of RNFLT, which provide evidence for the theory that the cerebral impaired glymphatic clearance may lead to neurodegeneration. Promoting glymphatic transport may be a new target for glaucoma. This study provides novel insights into the neurodegenerative processes underlying POAG and identifies the potential of multimodal neuroimaging for early disease detection and monitoring.

## Data Availability

The raw data supporting the conclusions of this article will be made available by the authors, without undue reservation.

## Footnotes

^1^ http://www.rfmri.org/

^2^ https://www.mathworks.com/

^3^ http://processmacro.org/

^4^ https://github.com
