# Supplementary material for: Diffusion tensor imaging along the perivascular space may reveal potential pathological mechanisms underlying disease progression in primary open-angle glaucoma patients
Source: Front Neurol. 2025 Oct 8;16:1659200. doi: 10.3389/fneur.2025.1659200 (PMC12540159; doi:10.3389/fneur.2025.1659200)
Supplement: Supplementary file 1 [file Table_1.docx]

**Supplementary materials**

**The specific steps of the method**

**Calculation of rs-fMRI indices of dynamics**

Five rs-fMRI indices were used in the concordance analysis, which were fALFF, ReHo, VMHC, DC, and GSC.

fALFF: ALFF was calculated by taking the mean amplitude of a time series in a specific frequency range (0.01- 0.1 Hz) following Fourier transformation[1], while the fALFF value was calculated as the ratio of low-frequency power spectrum (0.01-0.1 Hz) to the total power spectrum within the specified frequency range[2]. Due to the robust correlation observed between ALFF and fALFF, we selected fALFF for the concordance analysis due to its heightened sensitivity and specificity in identifying spontaneous brain activity[3].

ReHo: ReHo was employed to quantify the synchronization of neural activity within a local brain area over a given period, defined as Kendall's W coefficient of concordance between the time series of a particular voxel and those of its immediate neighbors (26 voxels)[4].

VMHC: The BOLD signal time-series Pearson correlation coefficient between particular voxels in the symmetrical areas of the left and right hemispheres was employed to determine the VMHC[5].

DC: DC is defined by the number of edges connected to a node in binary graphs or the total weight of the edges connected to a node in weighted graphs. In functional connectivity studies of the brain, a common method is to calculate the Pearson correlation coefficients between BOLD time series of various brain regions (or voxels) to obtain a grey matter functional connectivity matrix. The DC represents the cumulative count of connections exceeding a correlation coefficient of 0.25 throughout all its associated edges for each voxel[6].

GSC: The mean time series of all voxels within the group-level mask was first calculated by employing the GSC as the global signal. Subsequently, the Pearson correlation coefficient between the time series of each voxel within the group-level mask and the global signal was determined. The GSCorr value used in the subsequent calculations was the standardised score obtained after Fisher-Z transformation[7].

After these five types of rs-fMRI measures were completed, the Temporal Dynamic Analysis (TDA) toolkit, based on DPABI V6.0, was employed to examine the dynamics of these rs-fMRI indices[8]. A Hamming window with a window size of 50 TR and window stride of 1 TR was applied to segment the functional images of each participant, and a series of BOLD signal windows were obtained. Subsequently, these rs-fMRI metrics for each window were computed. To quantitatively characterise the temporal dynamic features of each index, the standard deviation (SD) of each index was calculated for each voxel over time in the whole brain. The SD maps were subjected to *Z*-score normalisation using a group mask for statistical analysis. Finally, the normalised SD maps of each dynamic rs-fMRI index were smoothed using a Gaussian kernel with a 4 × 4 × 4 mm^3^ full-width at half maximum (FWHM) in order to improve the signal-to-noise ratio.

**Fig S1 Analysis workflow diagram**


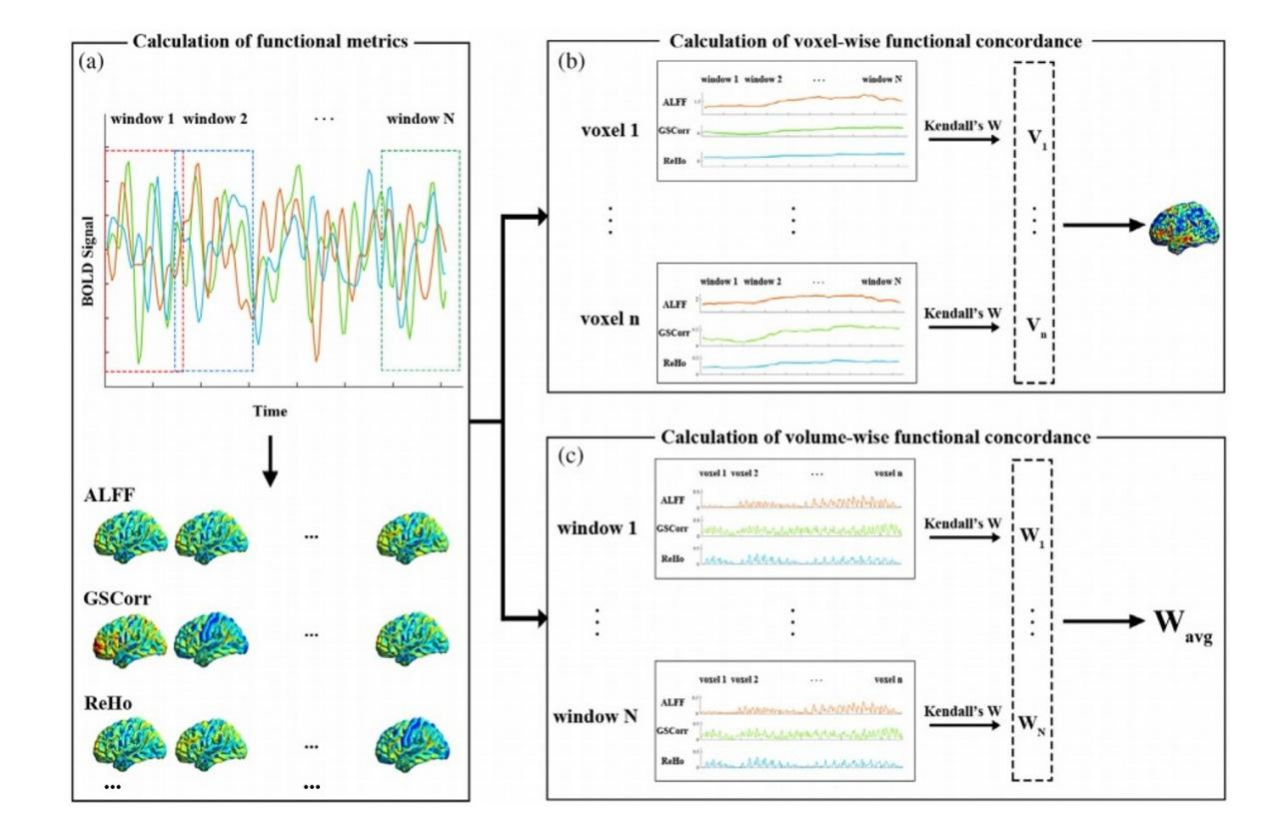


**Volume-wise and voxel-wise concordance**

Kendall's W method was used to assess volume- and voxel-wise concordance. This non-parametric statistic does not make any assumptions regarding the distribution and remains unaffected by the magnitude of disparities among the five dynamic rs-fMRI indices (including ALFF, ReHo, DC, VMHC, and GSC)[9]. Two types of concordance indices were calculated: 1) Volume-wise concordance index: first, for each window, the Kendall W value was computed for the five indices spanning across voxels. The average value of the volume-wise concordance for each participant was then calculated for all time windows and used as the basis for the results. 2) Voxel-wise concordance index: the voxel-wise concordance between the time windows for each participant was calculated. To facilitate subsequent analyses, a Gaussian kernel with an FWHM of 4 mm was used to smooth the voxel-wise concordance maps[10].

**MRI protocols**

MRI scans were obtained using a 3.0 T Siemens MAGNETOM Skyra scanner with a 32-channel head coil. fMRI images were recorded using an echo-planar imaging (EPI) sequence with the following parameters: repetition time (TR) = 2 s, echo time (TE) = 30 ms, flip angle = 90°, matrix size = 64×64, 35 slices, slice thickness = 3.4 mm, superior-inferior field-of-view (FOV) = 220 mm, and 240 volumes. Structural images were acquired using a three-dimensional (3D) magnetization-prepared rapid acquisition gradient echo (MPRAGE) sequence with TR = 2200 ms, TE = 2.48 ms, flip angle = 8°, matrix size = 256×256, 176 slices, voxel size = 0.9×0.9×1.0 mm³, and FOV = 230 mm. The T2-weighted protocol used a turbo spin-echo sequence with TR = 4000 ms, TE = 103 ms, flip angle = 150°, matrix size = 384×384, slice thickness = 6 mm (no gap), and FOV = 220 mm. DTI was conducted using a spin-echo single-shot EPI sequence with 64 diffusion-encoding directions, TR/TE = 8500/92 ms, flip angle = 90°, slice thickness = 2 mm, 58 slices, acquisition matrix = 128×128, FOV = 240 mm, and b-value = 1000 s/mm². During the scans, participants were instructed to keep their eyes closed, avoid cognitive tasks, and stay awake.

**DTI-ALPS-index calculation**

Diffusion-tensor images were preprocessed using DSI Studio graphic-user interface software version 10.15 (DSI Studio GUI; https://dsi-studio.labsolver.org/) . The process involved: (1) correcting phase distortion in DTI using reverse phase encoding (PE) volumes, followed by correction for eddy current and head movement artifacts, (2) generating a color-coded fractional anisotropy (FA) map and diffusive maps for the principal diffusion directions (x-axis: right-left or Dxx, y-axis: anterior-posterior or Dyy, and z-axis: inferior-superior or Dzz), and (3) applying both linear and non-linear transformations to align the FA map with the template space. The resulting transformation matrix was then utilized to align all the diffusive maps. Rectangular voxels (5mm×5mm) were placed at the lateral ventricle body level in projection and association fiber regions. Fiber orientation and diffusivities in the x-, y-, and z-axes were extracted at the voxel level from these regions of interest (ROIs). The DTI-ALPS index was calculated as the ratio of mean diffusivities along the x-axis in the projection (Dxxproj) and association fibers (Dxxassoc) to mean diffusivities along the y- and z-axes in the projection (Dyyproj) and association (Dzzassoc) fibers:

$$DTI-ALPS index=\frac{mean (Dxxproj,Dxxassoc)}{mean (Dyyproj,Dzzassoc)}$$

Two neuroradiologists (X.Z. and Z.W. with 5 and 8 years of experience, respectively) independently placed the ROIs. The overall glymphatic function of the participants was assessed using the average bilaterally calculated ALPS-index. For each participant, a neuroradiologist independently placed the ROIs and calculated the DTI-ALPS index independently. The mean of the results obtained by the two neuroradiologists was determined as the final result for each participant.

**Quantitative measurement of PVS volume**

PVS volume evaluation followed previously established protocols[11]. PVS volumes were quantitatively analyzed across three brain regions, specifically within the basal ganglia (BG), centrum semiovale (CSO), and the lateral ventricle body (LVB), using axial T2-weighted imaging. Two experienced neuroradiologists (X.Z. and Z.W.) performed the measurement using ITK-SNAP software (version 3.8; <http://www.itksnap.org/>), and their results were averaged to derive the final values for each participant, ensuring inter-rater reliability. The PVS volume for each region (BG-PVS, CSO-PVS, and LVB-PVS) was automatically calculated by summing the delineated PVS volumes. The PVS volume fraction was defined as the ratio of the PVS volume to the total volume of GM and WM. GM and WM segmentation was performed using the CAT12.8.1.

[1] Y.F. Zang, Y. He, C.Z. Zhu, Q.J. Cao, M.Q. Sui, M. Liang, L.X. Tian, T.Z. Jiang, Y.F. Wang, Altered baseline brain activity in children with ADHD revealed by resting-state functional MRI, Brain Dev 29(2) (2007) 83-91.

[2] Q.H. Zou, C.Z. Zhu, Y. Yang, X.N. Zuo, X.Y. Long, Q.J. Cao, Y.F. Wang, Y.F. Zang, An improved approach to detection of amplitude of low-frequency fluctuation (ALFF) for resting-state fMRI: fractional ALFF, J Neurosci Methods 172(1) (2008) 137-41.

[3] C.G. Yan, R.C. Craddock, X.N. Zuo, Y.F. Zang, M.P. Milham, Standardizing the intrinsic brain: towards robust measurement of inter-individual variation in 1000 functional connectomes, Neuroimage 80 (2013) 246-62.

[4] Y. Zang, T. Jiang, Y. Lu, Y. He, L. Tian, Regional homogeneity approach to fMRI data analysis, NeuroImage 22(1) (2004) 394-400.

[5] X.N. Zuo, C. Kelly, A. Di Martino, M. Mennes, D.S. Margulies, S. Bangaru, R. Grzadzinski, A.C. Evans, Y.F. Zang, F.X. Castellanos, M.P. Milham, Growing together and growing apart: regional and sex differences in the lifespan developmental trajectories of functional homotopy, J Neurosci 30(45) (2010) 15034-43.

[6] X.N. Zuo, R. Ehmke, M. Mennes, D. Imperati, F.X. Castellanos, O. Sporns, M.P. Milham, Network centrality in the human functional connectome, Cereb Cortex 22(8) (2012) 1862-75.

[7] A. Hahamy, V. Calhoun, G. Pearlson, M. Harel, N. Stern, F. Attar, R. Malach, R. Salomon, Save the global: global signal connectivity as a tool for studying clinical populations with functional magnetic resonance imaging, Brain Connect 4(6) (2014) 395-403.

[8] C.G. Yan, X.D. Wang, X.N. Zuo, Y.F. Zang, DPABI: Data Processing & Analysis for (Resting-State) Brain Imaging, Neuroinformatics 14(3) (2016) 339-51.

[9] C.G. Yan, Z. Yang, S.J. Colcombe, X.N. Zuo, M.P. Milham, Concordance among indices of intrinsic brain function: Insights from inter-individual variation and temporal dynamics, Sci Bull (Beijing) 62(23) (2017) 1572-1584.

[10] F. Lou, J. Tao, R. Zhou, S. Chen, A. Qian, C. Yang, X. Zheng, B. Chen, Z. Hu, M. Wang, Altered Variability and Concordance of Dynamic Resting-State fMRI Indices in Patients with Attention Deficit Hyperactivity Disorder, Front Neurosci 15 (2021) 731596.

[11] Huang SY, Zhang YR, Guo Y, et al. Glymphatic system dysfunction predicts amyloid deposition, neurodegeneration, and clinical progression in Alzheimer's disease. Alzheimers. Dement. 2024;20:3251-3269.

**High-resolution T1 volumetric processing and imaging data analysis**

This study employed voxel-based morphometry (VBM), deformation-based morphometry (DBM), and surface-based morphometry (SBM) analyses using MATLAB (r2013b) with the computational anatomy toolbox 12.8.1 (CAT12.8.1, http://www.neuro.uni-jena.de/cat/), an extension of SPM12. The preprocessing steps followed the CAT12 standard pipeline with default settings.

This study integrated voxel-based morphometry (VBM), surface-based morphometry (SBM), and deformation-based morphometry (DBM) to analyze 3D MPRAGE images. In VBM, images were aligned to the anterior commissure, segmented into gray matter (GM), white matter (WM), and cerebrospinal fluid (CSF), normalized to MNI space using DARTEL (1.5 mm³ voxel resolution), smoothed with an 8-mm FWHM Gaussian filter, and total intracranial volume (TIV) was derived from GM, WM, and CSF sums. SBM employed a projection-based method to assess cortical thickness, sulcal depth, cortical complexity (via fractal dimension), and gyrification index (pial-to-hull surface area ratio), with hemispheric surface reconstruction merged into a unified mesh and smoothed using 15-mm (thickness) or 20-mm (other metrics) FWHM Gaussian kernels. DBM transformed VBM deformation fields into Jacobian-based maps, quantifying voxel-wise expansion or shrinkage (|J| - 1) at 1.5 mm³ resolution, smoothed with an 8-mm FWHM Gaussian filter. These approaches provided a comprehensive analysis of brain morphology.

(1)VBM analysis

Initially, the 3D MPRAGE images were manually adjusted to align with the anterior commissure to improve registration accuracy. A non-linear deformation field was then estimated to align the tissue probability maps as accurately as possible with the images of the participants. The 3D MPRAGE images were segmented into gray matter (GM), WM, and CSF. The segmented data were normalized using the diffeomorphic anatomical registration through the exponentiated Lie algebra toolbox (DARTEL) to the standard MNI space, with an isotropic voxel resolution of 1.5×1.5×1.5 mm³. An 8 mm full width at half maximum (FWHM) Gaussian filter was used to facilitate statistical analysis. Subsequently, the total intracranial volume (TIV) was estimated for each participant by summing GM, WM, and CSF volumes[12].

(2)SBM analysis

A projection-based approach was used to estimate the distance between the inner and outer cortical surfaces for cortical thickness. These surfaces demarcated the boundaries between WM and GM, as well as between GM and CSF. Additionally, three geometric measures were calculated: sulcal depth, cortical complexity, and degree of gyrification. Sulcal depth represents the depth of grooves or fissures on the brain surface, reflecting the folding pattern of the cerebral cortex. Cortical complexity, quantifying the cortical folding pattern, was measured using the fractal dimension. The degree of gyrification, which measures the cortical complexity and folding, was computed using the mean surface curvature of the brain as previously described[13]:

$$Gyrification index=\frac{area of local pial surface}{area of local hull surface}$$

Each hemisphere underwent individual surface reconstruction using the projection-based thickness approach. This technique corrects surface mesh defects and accounts for spherical inflation and registration to ensure accurate topology. A modified DARTEL algorithm was used during spherical registration to project the matrix onto a standardized spherical surface for inter-participant analysis. The meshes from the right and left hemispheres were merged to create a unified mesh. Cortical thickness images were smoothed using a 15-mm FWHM Gaussian kernel, while other parameters were smoothed using a 20-mm FWHM Gaussian kernel.

(3)DBM analysis

For each participant, 3D MPRAGE images were transformed into DBM maps following a previously described procedure[14]. The non-linear transformation obtained from the VBM analysis was inverted to derive deformation fields that mapped voxel coordinates (χ1, χ2, χ3) from the participant's native space to corresponding voxels in the MNI template [u1(χ); u2(χ); u3(χ)]. Jacobian matrices of the deformation were generated and estimated using a first-order approximation. Voxel-wise relative deformation values (|J| - 1) were calculated to create the DBM maps, indicating the expansion or shrinkage of brain voxels during registration to the MNI template. The data were resampled to a voxel size of 1.5×1.5×1.5 mm³ and smoothed using an 8-mm FWHM Gaussian filter statistical analysis.

[12]. Ge X, Wang L, Pan L, et al. Alteration of the cortical morphology in classical trigeminal neuralgia: voxel-, deformation-, and surface-based analysis. *J Headache Pain* 2023; **24**(1): 17.

[13]. Luders E, Thompson PM, Narr KL, Toga AW, Jancke L, Gaser C. A curvature-based approach to estimate local gyrification on the cortical surface. *Neuroimage* 2006; **29**(4): 1224-30.

[14]. Tremblay C, Abbasi N, Zeighami Y, et al. Sex effects on brain structure in de novo Parkinson's disease: a multimodal neuroimaging study. *Brain* 2020; **143**(10): 3052-66.

**Comparison of PVS volume fraction between patients with POAG and HCs (Figure S2-S7)**

In patients with POAG, the ICCs for BG-PVS, CSO-PVS, and LVB-PVS were 89.1% (95% CI: 0.86–0.93, *P*<0.001), 92.5% (95% CI: 0.89–0.94, *P*<0.001), and 94.1% (95% CI: 0.90–0.96, *P*<0.001), respectively, compared to 95.5% (95% CI: 0.91–0.97, *P*<0.001), 91.2% (95% CI: 0.87–0.94, *P*<0.001), and 93.1% (95% CI: 0.89–0.95, *P*<0.001) in HCs (Figure S2-S7).

**Fig S2 BG-PVS volume fraction in POAG for both observers**

**

**

**Fig S3 LVB-PVS volume fraction in POAG for both observers**

**

**

**Fig S4 CSO-PVS volume fraction in POAG for both observers**

**

**

**Fig S5 BG-PVS volume fraction in HCs for both observers**

**

**

**Fig S6 LVB-PVS volume fraction in HCs for both observers**

**

**

**Fig S7 CSO-PVS volume fraction in HCs for both observers**





**Differences in ALPS-index between patients with POAG and HCs and visual field deficit severity subgroup analysis (Figure S8-S11)**

For the left ALPS-index (ICC=0.876, 95% CI: 0.85–0.93, P<0.001) and the right ALPS-index (ICC=0.865, 95% CI: 0.84–0.92, P<0.001) (Figure S8-S11), the inter-observer reliability was excellent in patients with POAG.

**Fig S8 ALPS index in the left hemisphere of POAG for both observers**

**

**

**Fig S9 ALPS index in the right hemisphere of POAG for both observers**

**

**

**Fig S10 ALPS index in the left hemisphere of HCs for both observers**

**

**

**Fig S11 ALPS index in the right hemisphere of HCs for both observers**

**

**
